# Modified Optimism

**Published:** 1921-09-10

**Authors:** 


### Modified Optimism.

The half-yearly meeting of the Leicester and County Saturday Hospital Society held at the Leicester Royal Infirmary indicated that, while the finances of the six months were difficult to review, the prospects for the future were not gloomy, and there was a distinctly optimistic feeling that things are on the eve of recovery. The Society is not experiencing that rush of applications for benefits which usually accompanies bad trade periods. The number of patients receiving convalescent benefit shows an increase of 142 in the half-year, and the expenses have increased by £1,395. This is partly due to a new installation of laundry machinery at the Swithland Home, which will do the washing of the new Children's Home as well. In connection with the latter Home the committee have recently acquired 5½ acres of additional woodland adjoining the Home, and the Society is now in possession of a compact block of property at Woodhouse Eaves of 17 acres of laid-out gardens, pleasure-grounds, and woodland. The Society has no inclination for State control either of the Infirmary or of the Homes, believing there are still great possibilities in the voluntary system of support. Mr. S. A. Gimson, in moving the adoption of the report, said what is now very much to be desired is some sort of co-ordination of all institutions and efforts in the county, so that there may be no danger of competition between institutions. The Leicester Royal Infirmary in its last annual report welcomed such a scheme and gave an important lead in the matter of co-ordination.

